# 胸腺肿瘤预后因素的现状

**DOI:** 10.3779/j.issn.1009-3419.2014.02.11

**Published:** 2014-02-20

**Authors:** Frank Deterbeck, Samuel Youssef, Enrico Rufni, Meinoshin Okumura, 北京大学肿瘤医院 付浩

**Affiliations:** 1 Yale University School of Medicine, New Haven, Connecticut; 2 Department of Toracic Surgery, University of Torino, Torino, Italy; 3 Department of General Toracic Surgery, Osaka University Graduate School of Medicine, Osaka, Japan

当患者被诊断某种疾病后，患者最想知道的信息就是该疾病的预后。预后受多种因素的影响，而对于肿瘤而言，最重要的因素是肿瘤累及的解剖范围，这就是肿瘤分期。分期系统不应与预后相混淆，肿瘤分期仅为预后的一部分内容。预后还会受治疗、患者个体因素（如合并症）、肿瘤特异相关因素和其他因素的影响，这些都参与构成预后因素。目前，ITMIG联合IASLC在UICC和AJCC的领导下，正在着手制定一个实用的正式的分期系统。制定解剖学分期系统的一个必然而重要的环节是评估预后因素。通过回顾文献寻找有用的预后因素是这一过程的第一步。

一般来说，对预后因素进行研究和分类是最基本的。但是人们常常存在疑惑，因为不清楚何种因素与结果有关，如一个因素可能与总生存有关，可能与疾病是否治愈有关，也可能与是否有治疗反应有关。在特殊情况下，预后因素可能是特异性的，如某因素只是手术治疗的胸腺瘤患者的预后因素，而另一个因素则是放化疗患者的预后因素。预后因素可以分为几类：肿瘤相关因素，也就是最主要的因素，患者个体因素，也就是即使没有肿瘤，这些因素也存在，和环境因素，比如能否获得最佳治疗之类的事情。一旦我们确定了某个预后因素，我们会立即开始试图把结果改变为我们想看到的那样（检验该预后因素的的真实性）。最后，需要理解，在确定预后因素的过程中，存在很多统计学陷阱和错误影响预后因素的准确性，这在另一篇文章中讨论^[1]^。由于对判断预后因素的经验存在不足，故急需在此方面投入研究。本文就目前胸腺瘤预后因素的现状作以下综述。

## 1 方法

检索1980-01-01至2010-12-31发表的关于胸腺瘤或胸腺癌的生存或复发的英文文献。并追加检索每篇被检文献后面的参考文献、近期专著的相关章节和综述文献、和作者自己查找的独立文献。除了总生存和复发，我们没有评估其他结局的预后因素，主要是因为仅有几个单中心研究。

对胸腺恶性肿瘤而言，复发是评估预后最好的方法，这在近期ITMIG的一篇具有里程碑意义的文章中有讨论。遗憾的是，几乎没有研究对此加以强调。关于复发的分析我们纳入了无疾病生存的概念，尽管将复发和死亡视为平等的研究终点存在一定问题，因为容易得到，所以总生存是最常用的研究终点。但是，它不是一个理想的研究终点，因为很多胸腺瘤患者死于其他原因，即使复发其存活时间也较长^[2]^。不同的研究使用不同的方法，例如总生存和胸腺瘤特异性生存，这将得出不同的生存结果。但是，因为分析的目的是评估不同因素的预后价值，因此有必要利用不同评估方法来提高结论的可靠性。一个良好的预后因素必须具有独立意义，而且必须评估与其他因素的交互作用。正因为如此，本文仅纳入多因素分析的研究。多因素分析的方法和分析因素的因素数目或特征不限。仅纳入病例数大于等于75例的研究。因为经过粗略估算至少需要75例患者，才能使3个中等效应的因素达到80%的检验效能^[2]^。

本文纳入研究报道的病例范围很广泛，多数病例为接受手术治疗者，也包括了除手术外还有辅助治疗的患者。为了评估这些患者，加入了不同治疗方式的比例（如表所示）。纳入研究中未作亚组分析，部分因为各研究的方法不同，同时也因为样本量较少，达不到统计分析量。此外，报道特殊病例组的研究数量有限，得不出任何结论，因而未纳入分析。另有几个研究包括了不同治疗类型的患者；如上两种情况我们只选用其中的手术结果。本文排除了重复的文献，或者同一单位先后两篇一个样本量小一个样本量大的文章。但是本文纳入了同是一个单位的病例，虽有重叠但每篇中均有不同特征的病例的文章。本文将研究的数据提取列表，并将在多因素分析中*P*值小于等于0.05的因素记录为阳性因素^[2]^。因为多因素分析的方法不同，可能会有较高的假阳性率。例如，一个变量被分为两组选取了最好的界值作分界线，从而导致统计学上过度乐观的结果，称之为“双向挖掘”^[1, 2]^。我们将这些有高假阳性风险的研究列表分析，用这样的方法是不能分析实际风险的。

对于样本量有限的研究，因其检验效能低，可出现假阴性结果。如果报道的因素P值大于0.05，该因素记录为阴性。根据研究样本量、分析因素的数量和差异的大小能够进行粗略的估算。我们对研究的阴性结果进行了先验估计，这种粗略的评估达到中等效应至少需要80%的检验效能。最后，我们发现所有大于75例患者的研究都符合这一要求。

不是所有的研究都分析同样的因素，有些重要因素漏评会严重影响结果，但是本文并没有试图对此纠正，因此，我们尽可能全面的搜集了数据。报道中记录为阳性、阴性或未评估的数据，提供了预后因素的总体概况。此外，很多报道选择二分变量方法，但是选择的分界值又各不相同，本文努力综合了这些界值相似的数据，并在表格下方作了注释。

一些研究报道了不同的多因素分析，例如，有些包含而另一些不包含一些因素，或使用不同的定义的研究。因为缺乏判断标准，本文将这些研究分别独立的列在表中。另外，在一些研究对某些主要因素作了多因素分析以便肯定某一因素的影响或其与其他因素的关联^[3-8]^。这似乎能加强MVA的目的以便找出哪些因素真正影响生存，而哪些又是偏倚造成（假阳性）。在这些研究分析中没有包含所有研究因素时，我们收集其子集分析数据（例如，有分期没有组织学，有组织学没有分期）。通过收集所有的子集分析，希望呈现出一个较少偏倚的整体概况。然而，这也说明研究过程中会存在潜在的偏倚。本文排除了其中一项研究，因为该研究在分析每个因素时，去除了其他具有潜在意义的因素。同时因为这个研究没有报道整体的多因素分析，虽然其结果存在偏倚，甚至对于哪个因素加入或去除都没有详细报道。

总结数据时，本文计算了具有显著差异的多因素研究的比例。对此应该非常谨慎的解释，因为这篇文献中可能有我们没有发现的许多假阳性和假阴性结果。对于少于5篇总生存研究或少于3篇复发研究中分析的因素，不列在表中，因为从这些有限的数据中不能得出有意义的结论。

## 2 结果

按照上述检索和纳入标准，共纳入29篇有关生存预后因素的多因素研究和12篇复发预后因素的多因素研究。少数研究中既包括生存预后又包括了复发预后因素的分析^[4, 8, 9]^，按照方法部分的描述将每种分析列在表中。结果见表 1和表 2，图 1和图 2。

**1:** 预后因素多因素分析 Multivariate Analysis of Factors Predicting Better Survival

	治疗（%）						良好预后因素						
Study	n	R_0_	Ch	RT	Stage Ⅰ, Ⅱ	R_0_	Hist Thym	Hist w/TC	Older Age^a^	Small Size	Male Gender	MG	No.of Additional factors
强稳健统计学方法的研究													
Ruffini 10^10b^	255	87	2	45	0.001	—	NS	—	—	—	—	NS	—
de Jong 08^11c^	232	41	10^d^	33^d^	0.01	—	—	＜0.001	＜0.001^e^	—	NS	NS	1
Rieker 02^6^	218	77	14	39	＜0.001	—	—	＜0.03	NS	NS	NS	NS	2
Park 04^12^	150	69	—	—	＜0.001	—	—	＜0.02	NS	—	NS	NS	—
Venuta 97^13b^	148	—	33^d^	—	0.0001	—	NS^f^	—	0.001	—	—	—	2
Park 04^12^	133	77	—	—	0.006	NS	—	NS	NS	—	＜0.04	NS	—
Kim 05^7g^	108	82	16	28	＜0.03	NS	—	NS	NS	NS	NS	NS	—
弱稳健或不明确统计方法的研究													
Kondo 03^14b^	1093	—	—	—	＜0.001	＜0.001	NSf	—	NS	—	NS	NS	3
Margaritoria 10^15^	317	93	—	38	NS	0.001	—	NS	NS	—	NS	NS	2
Regnard 96^9^	307	85	6	52	NS	0.00001	—	NS^f^	—	—	—	NS	—
Lewis 87^16b, g^	283	83	2	26	＜0.05	＜0.05	NS^f^	—	＜0.05	NS	NS	NS	5
Regnard 96^9^	260	100	6	52	0.00001	—	—	NS^f^	—	—	—	NS	—
Okumura 02^17b, g^	243	95	10^d^	60^d^	＜0.001	NS	0.05	—	NS	—	NS	NS	1
Ströbel 04^18g^	228	67	17	32	＜0.05	＜0.05	—	＜0.05	NS	NS	NS	NS	1
Fang 05^19^	204	88	—	—	＜0.001	0.004	—	0.001	—	—	—	NS	—
Lee 07^20^	195	83	5	40	＜0.001	NS	—	＜0.001	NS	—	NS	NS	—
Rena 05^21b^	178	84	13	43	＜0.04^d^	＜0.02	＜0.03	—	NS	—	NS	NS	1
Cowen 95^22b, h^	149	42	50	100	NS	(0.003)^i^	—	—	0.013	0.001^j^	—	NS	1
Wilkins 99^23^	136	68	7	37	NS^k^	＜0.001	—	.0.02^f^	0.036^e^	—	NS	0.005	4
Nakagawa 03^4b, g^	130	95	4	5	＜0.01	NS	—	—	NS	0.01	NS	NS	—
Nakagawa 03^4b, g^	130	95	4	5	—	0.002	0.01	—	NS	0.001	NS	NS	—
Rea 04^24^	132	82	18	47	0.003	NS	—	0.0001	NS	—	NS	NS	2
Lucchi 09^25b, l^	123	95	17	73	0.04^m^	—	NS	—	NS	NS	NS	—	2
Blumberg 95^26^	118	73	32	58	0.003	0.0006	—	0.004^f^	NS	0.0001	NS	NS	—
Pan 94^27b^	112	80	—	—	＜0.05	—	NS	—	—	—	—	—	—
Quintanilla 94^28b^	105	100	0	24	＜0.05	—	＜ 0.05^f^	—	NS	NS	NS	NS	1
Zisis 05^8b^	104	100	14	63	—	—	0.05	—	NS	NS	—	NS	2
Zisis 05^8b^	104	100	14	63	＜0.05	—	—	—	＜0.02^e^	—	NS	NS	2
Kondo 04^29^	100	84	28	37	0.04	＜0.05	—	NS	NS	—	NS	NS	—
Kim 10^30n^	100	79	7	67	NS	—	NS	—	—	NS	—	—	—
Kim 08^31h^	100	78	45	100	0.04	NS	—	0.02	＜0.03	—	—	NS	—
Chalabreysse 02^5^	90	67	3	12	—	—	—	＜0.001	NS	—	—	NS	—
Rieker 07^32^	77	74	30^d^	62	NS	0.001	—	0.001	NS	—	NS	NS	1
总结：阳性%^a^					83%	63%	42%	67%	15/11%^P^	36%	4%	3%	
纳入标准：1980年-2010年，病例数≥75例的预后多因素研究。排除少于5项研究评估的因素。使用“双向数据挖掘”的“弱稳健统计学方法”的研究有得出假阳性结论的风险。 ^a^年龄界限不同：＞30岁（Lewis, Cowen）、＞60岁（Venuta）、＞57岁（Wilkins, Kondo, Rieker）、＞52岁（Rena）或不确定。^b^排除胸腺癌; ^c^队列研究；^d^评估值，非特异性报告；^e^在本组研究中，老年组预后更差；^f^非WHO组织分类；^g^胸腺瘤特异生存；^h^以放疗患者为基础的研究；^i^单纯活检与切除比较；^j^定义为肿瘤无纵隔内压迫；^k^比较Ⅰ期与Ⅱ-Ⅳ^l^MG患者为基础的研究；^m^定义不明确；^n^仅针对B型胸腺瘤；^o^排除括号内数值；^p^15%与良好预后相关，11%与不良预后相关；Ch，化疗；Hist，组织类型；MG，重症肌无力；NS，无统计学意义；R0，完全切除；RT，放疗；w/TC，包括胸腺癌；Thym, 胸腺瘤。 注：本表已获得版权所有者© 2011 by the International Association for the Study of Lung Cancer复制许可。													

**2:** 复发率或无疾病生存预后因素的多因素研究 Multivariate Analysis of Factors Predicting Lower Rates of Recurrence or Disease-Free Survival

		治疗（%）				低复发率预测因素								
	*n*	%R0	Ch	RT		Stage Ⅰ, Ⅱ	R0	Hist Thym	Hist w/TC	Older Age	Small Size	Gender	MG	No.of Additional factors
强稳健统计学方法的研究														
Ruffini 1010a, b	255	87	2	45		0.001	—	NS	—	—	—	—	NS	—
Rieker 026a	218	77	14	39		＜0.05	—	—	＜0.05	NS	NS	NS	NS	2
Wright 0533	179	90	—	—		＜0.0001	NS	—	0.003	NS	0.001	NS	NS	3
Huang 0934	112	73	67	43		(NS)C	＜0.001	—	0.006	NS	NS	NS	—	2
弱稳健或不明确统计方法的研究														
Margaritoria 1015a	317	93	—	38		＜0.001	NS	—	NS	NS	—	NS	NS	2
Margaritoria 1015a	295	100	—	36		＜0.001	—	—	0.003	NS	—	NS	＜0.0001	2
Rena 0521a, b	178	84	13	43		0.01	0.0001	＜0.02	—	NS	—	NS	NS	1
Cowen 9522b.d	149	42	50	100		0.04	(0.003)e	—	—	0.006	0.001f	—	NS	1
Blumberg 9526	118	73	32	58		0.03	NS	—	NS	NS	NS	NS		—
Quintanilla- Martinez2a, b	105	100	0	24		0.03	—	0.03g, h	—	NS	NS	NS	NS	1
Kondo 0429a	100	84	28	37		0.002	0.05	—	NS	NS	—	NS	NS	—
Kim 1030a, b, i	100	79	7	67		0.002	—	NS	—	—	0.002	—	—	—
Kim 0831a, d	100	78	45	100		0.04	NS	—	0.01	NS	—	NS	NS	3
总结：阳性%^a^						100%	43%	50%	63%	9%	43%	0%	9%	
纳入标准：1980年-2010年，病例数≥75例的预后多因素研究。排除少于3项研究评估的因素。使用“双向数据挖掘”的“弱稳健统计学方法”的研究有得出假阳性结论的风险。 a无疾病生存；b排除胸腺癌；cⅢ期与Ⅳ期对比；dRT患者为基础的研究；e单纯活检与切除对比；f定义为肿瘤无纵隔内压迫；g非WHO组织分类；h与分化好的胸腺癌相比（分析排除未分化胸腺癌），髓质型、混合型或皮质型复发率更低；i仅针对B型胸腺瘤；j排除括号内数值。 Ch，化疗；Hist，组织类型；MG，重症肌无力；NS，无统计学意义；R0，完全切除；RT，放疗；w/TC，包括胸腺癌；Thym，胸腺瘤。 注：本表已获得版权所有者© 2011 by the International Association for the Study of Lung Cancer复制许可。														

**1:**
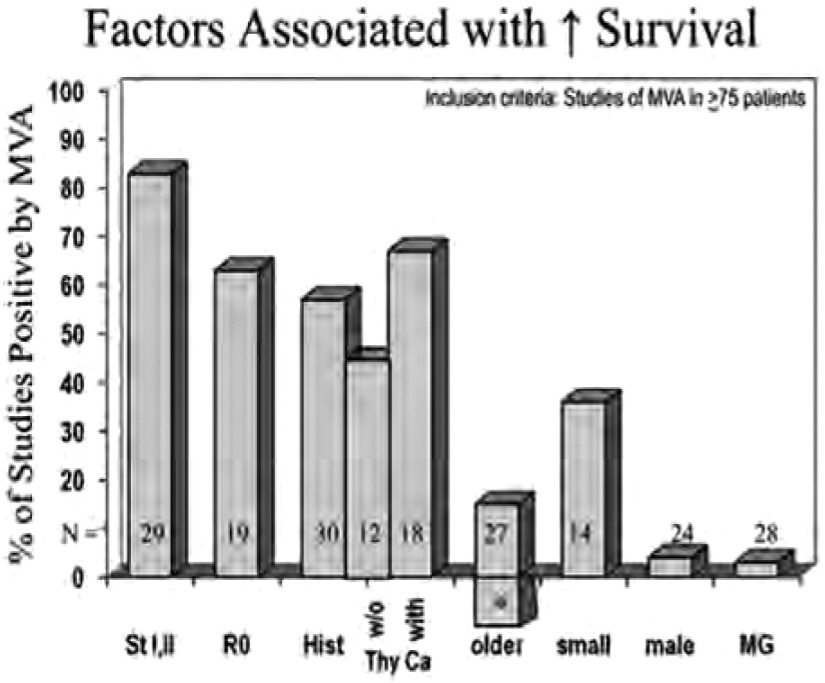
生存相关因素的多因素分析。提示为阳性预后因素研究占纳入多因素分析（1980-01-01至2010-12-31，病例数＞75例的研究）的百分比。^*^提示老年患者生存差的研究的比例。Hist, 组织类型；MG，重症肌无力；MVA，多因素分析；N，验证该因素的研究数量；St，分期；Thy Ca，胸腺癌；w/o，无。 Factors associated with increased survival by multivariate analysis. Percentage of studies finding a factor prognostically significant for survival in multivariate analysis in studies of 75 patients from January 1, 1980, to December 31, 2010. ^*^Percentage of patients showing that older patients had worse (not increased) survival. Hist, histologic typing; MG, myasthenia gravis; MVA, multivariate analysis; N, number of studies examining this factor; St, stage; Thy Ca, thymic carcinoma; w/o, without.

**2:**
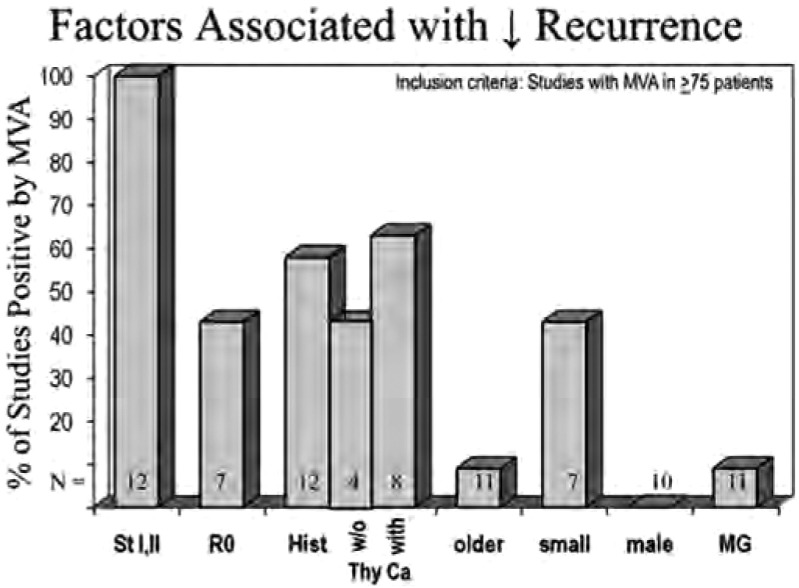
复发相关因素的多因素分析。提示为复发或无疾病生存良好预后因素的研究占纳入多因素分析（1980-01-01至2010-12-31，病例数＞75例的研究）的百分比。Hist, 组织类型；MG，重症肌无力；MVA，多因素分析；N，验证该因素的研究数量；St，分期；Thy Ca，胸腺癌；w/o，无。 Factors associated with decreased recurrence by multivariate analysis. Percentage of studies finding a factor prognostically significant for recurrence or disease-free survival in multivariate analysis in studies of75 patients from January 1, 1980, to December 31, 2010. Hist, histologic typing; MG, myasthenia gravis; MVA, multivariate analysis; N, number of studies examining this factor; St, stage; Thy Ca, thymic carcinoma; w/o, without.

在多数研究中并没有患者的详细资料，但可被粗略看成是为了接受根治性治疗，关于接受治疗的数据总结在表中。大多数患者接受手术治疗，约一半的患者接受了放疗和约25%的患者接受的化疗。其中值得一提的是：de Jong^[11]^作的队列研究，Cowen^[22]^报道中患者仅接受了放疗，Lucchi^[25]^报道的所有患者都患有重症肌无力。

对于复发和生存均有确切显著性意义的因素是分期，多数研究是比较分期Ⅰ期+Ⅱ期与Ⅲ期+Ⅳ期的治疗。多数研究都采用Masaoka或Masaoka-Koga分期，事实上，在所有的分期系统中，这种二分法产生基本相同的两组患者。应该明确说明的是，这些多因素分析中的大多数没有明确Ⅰ期与Ⅱ期，Ⅱ期与Ⅲ期，Ⅲ期与Ⅳa期之间预后的差别，或是从Ⅰ期到Ⅳb期之间预后连续的变化的过程^[35]^。但是，多项研究表明复发和生存逐渐变差，尽管存在是否Ⅰ期和Ⅱ期之间有预后差异的问题。尽管这些预后因素研究存在局限性，但是不难看出分期是有用的预后因素。

另一个对于复发和生存相对肯定的预后因素是完全切除，很明显这只能用于手术治疗的亚组患者。尽管完整切除率与肿瘤的分期明确相关，但是经多因素分析得出R0切除似乎是具有独立意义的预后因素^[35]^。

胸腺恶性肿瘤的组织亚型也是很重要的预后因素，但很难全面评估。首先，组织分类方法不统一，尽管近来更多的使用了WHO分类系统^[37, 38]^。其次，病理医生对于组织分类的标准有差异^[39-41]^。最大的问题是多数研究只报道了组织分型的预后价值，而没有说明具体组织亚型之间的差异。此外，在多数研究中二分法的使用，首先选择最大化差异，之后进行显著性检验（即，双向挖掘）。在这些研究中最佳二分法使用了很多不同的分界值，这使得其结果可信度降低。胸腺癌似乎始终与最差的生存相对应，但是还不能得出是否是独立的预后因素。本文试图用多因素分析的方法在包含和不包含胸腺癌的组群中对此进行探索，结果表明包含有胸腺癌的研究与仅包含胸腺瘤的研究相比，总生存之间有差异（67%比42%）。但是，在评估复发的研究中没有发现差异。

年龄因素影响很小，年龄似乎不影响复发。有人可能会作出这样的预言，如果年龄对复发没有影响，那么预测老年患者的生存会更差，因为老年人死于其他原因的风险增加。但是，几项研究已经发现对于总生存，年龄是一个良好的预后因素，也有相当的研究认为它是一个不良预后因素。但是这些研究中年龄的分界值选取不同。鉴于这样矛盾的结果，最好不要将年龄视为有效的预后因素。

在少数研究中，发现体积小的肿瘤是复发和生存良好的预后因素。性别和重症肌无力似乎对复发或生存不具有预后意义。

仅少数文献对其他因素作了研究。有关生存因素包括：时间段^[11, 13, 15, 16]^，复发的出现与否^[8, 18, 24, 31]^，是否有重症肌无力之外的副瘤综合征^[14, 16, 21, 24]^，症状轻重^[16, 23]^，是否有合并症^[6, 15]^，淋巴样增生与否^[6, 28]^，重症肌无力的缓解与否^[8, 42]^，辅助放疗与否^[9, 42]^，辅助化疗与否^[22]^，放疗剂量大小^[31]^，是否有第二原发肿瘤^[23]^，是否侵犯大血管^[17]^，是否侵犯胸膜^[31]^，是否淋巴结转移^[14]^，是否远处转移^[14]^，重症肌无力的分级和多学科治疗的顺序^[42]^，术前是否活检^[23]^，细胞异型性与强度指数^[16]^，患者PS评分^[32]^。所有这些研究因素均未发现有预后意义，但发现下列情况，4篇研究时间段的文献中有1篇表明有预后意义^[13]^，4篇研究复发时间的文献中有1篇表明有预后意义^[31]^，2篇研究重症肌无力缓解的文献中有1篇表明具有预后意义^[42]^，还有1篇认为大血管侵犯有预后意义^[17]^。

其他被研究的因素有（3篇以内）：复发时间^[15, 33]^，术前治疗状态^[34]^，辅助化疗与否^[22]^，多学科治疗顺序^[31]^，放疗剂量大小^[31]^，是否有合并症^[6, 15]^，除重症肌无力之外的副瘤综合征^[21, 33]^，淋巴样增生与否^[6, 28]^，血管或周围结构侵犯与否^[33]^，胸膜侵犯与否^[31]^，和种族^[34]^。除了胸膜侵犯外（仅一篇研究支持），所有这些因素均不具有预后意义^[31]^。

总之，目前还没有整合好的实用的预后系统，大家正在思考建立这样一个系统。尽管检验预后因素有效性的统计学方法有了很大发展，但尚未被广泛认可。由于急需胸腺瘤的预后判断系统，预后因素的定义不能等到所有的框架构成后才确立。但是，我必须认识到定义预后因素是一个演变的过程，对目前已有的这些预后因素要持怀疑态度。

本文试图说明现有研究中统计分析的不足，以便在前瞻性研究中避免，但非常困难。总的来说，在我们获得的这些研究中，大多数使用的方法带有得出假阳性预后因素的风险，这在更严格的分析中不能重复。分析发现，在有相似结果的研究中多少包含有强有力的统计方法。关于假阴性结果，多是由于试图利用有限的数据评估大量的因素所造成的，我们没有发现任何报道分析有非常有限的检验效能。但是，需要注意的是，报道研究的样本仅允许检验具有中度或高度效应的预后因素；任何因素都不能排除对预后有低度效应表现。在这些研究中，很多时间跨度有20年甚至更长。仅有少量研究把治疗时间段作为预后因素，分析其效应，仅一项研究发现有统计学意义^[11, 13, 15, 16, 33]^。随着时间变化的临床实践和胸腺恶性肿瘤各方面定义的不同，导致了很多含糊不清问题，这迫使我们对现有预后因素需要从根本上加以认识。

现有数据表明，分期和完全切除可作为有效的预后因素。性别和重症肌无力表现没有预后意义。肿瘤大小的预后意义需要进一步研究，大小的分界值选择存在一些问题，不规则肿瘤大小如何测量也存在问题。肿瘤组织分类的影响也需要进一步研究。一些单中心研究中的生存曲线一致表明胸腺癌有着更差的预后，因此，这一病理类型在所有的分期系统都被特别关注。胸腺癌预后差的一致事实表明组织分类具有预后意义，但何时需要排除这些肿瘤尚不清楚。关于胸腺癌的组织学分类问题目前正在讨论。因此，在明确其他组织类型是否具有预后意义之前，暂不将胸腺癌视为独立的预后因素，其预后意义有待于进一步的研究。

由于胸腺肿瘤生长较慢的生物学行为，将复发作为评估预后的方法要优于总生存。一般来讲，关于复发预后因素的数据（表 2）与总生存的数据是平行的（表 1）。因此大多数研究使用无疾病生存作为研究终点，这至少可以部分的解释类似的结果。把复发和死亡视为等同的研究终点是不明智的。仅有4项研究将重点集中在复发^[22, 26, 33, 34]^，这显然是要在将来预后研究中必须解决的问题。
